# Methyltransferase like 13 mediates the translation of Snail in head and neck squamous cell carcinoma

**DOI:** 10.1038/s41368-021-00130-8

**Published:** 2021-08-11

**Authors:** Xiaochen Wang, Kang Li, Yuehan Wan, Fangfang Chen, Maosheng Cheng, Gan Xiong, Ganping Wang, Shuang Chen, Zhi Chen, Jianwen Chen, Xiuyun Xu, Cheng Wang, Liang Peng, Demeng Chen

**Affiliations:** 1 Center for Translational Medicine, Institute of Precision Medicine, The First Affiliated Hospital, Sun Yat-sen University, Guangzhou, China; 2 Department of Oral and Maxillofacial Surgery, Hospital of Stomatology, Guanghua School of Stomatology, Sun Yat-sen University, Guangzhou, China; 3 Oncology Department, Chinese PLA General Hospital, Beijing, China

**Keywords:** Head and neck cancer, Prognostic markers

## Abstract

Methyltransferase like 13 (METTL13), a kind of methyltransferase, is implicated in protein binding and synthesis. The upregulation of METTL13 has been reported in a variety of tumors. However, little was known about its potential function in head and neck squamous cell carcinoma (HNSCC) so far. In this study, we found that METTL13 was significantly upregulated in HNSCC at both mRNA and protein level. Increased METTL13 was negatively associated with clinical prognosis. And METTL13 markedly affected HNSCC cellular phenotypes in vivo and vitro. Further mechanism study revealed that METTL13 could regulate EMT signaling pathway by mediating enhancing translation efficiency of Snail, the key transcription factor in EMT, hence regulating the progression of EMT. Furthermore, Snail was verified to mediate METTL13-induced HNSCC cell malignant phenotypes. Altogether, our study had revealed the oncogenic role of METTL13 in HNSCC, and provided a potential therapeutic strategy.

## Introduction

Human head and neck squamous cell carcinoma (HNSCC) is one of the most diagnosed malignancies worldwide with high mortality and morbidity, making it a growing health burden^1,2^. And this highly malignant disease is characterized by poor prognosis due to its high local recurrence and lymph node metastasis^3^. As for the metastasis of malignant tumor, epithelial–mesenchymal transition (EMT) was a crucial part^4,5^. It was activated in tumor progression^6,7^ and affected cancer stem cell (CSC) cellular behaviors^8,9^. During EMT, a small subset of cancer cells acquires motility with decreased adhesive ability and obtains invasive properties. Several EMT-induced transcription factors, such as Slug, Twist, and Snail, have been involved in the progress^10^. Specifically, Snail, a major transcription factor governing EMT, equips cancer cells with malignant phenotypes such as tumor recurrence and metastasis. Furthermore, although many of the signaling pathways could impinge on the transcriptional regulation of the Snail gene^11^, posttranscriptional mechanisms affecting Snail are also emerging as crucial regulators of its activity^12^.

Especially, aberrant regulation of mRNA translation to protein often accounts for the progression of cancer^13^. For instance, the subunits of the eukaryotic translation initiation factor 4F complex and eukaryotic elongation factor 2 were frequently found to be dysregulated in multiple cancer types^14,15^. Increased activities of translation regulators enable the cancer cells to rapidly produce selective proteins in response to cancer development. For example, eukaryotic translation elongation factor 1 alpha 2 can mediate the expression of EMT factor matrix metallopeptidase 9 (MMP9) in pancreatic cancer^16^. In addition, chemical modification of translation regulators by phosphorylation, acetylation, methylation, and ubiquitination can result in changes of translation efficiency. For instance, phosphorylation of eIF2a leads to the gene-specific translation, which further support cell survival in human cancers^17,18^.

Recently, studies reported that human methyltransferase like 13 (METTL13) can catalyze demethylation of eukaryotic elongation factor 1A (eEF1A) lysine 55 (eEF1AK55me2) to promote the protein synthesis in Ras-driven cancers^19,20^. METTL13 was characterized with oncogenicity, as it was involved in various diseases, such as gastric cancer^21^, lung cancer^22,23^, and breast cancer^24^. METTL13 also functions as a potential inhibitor of apoptosis, for it was found that miR-16 promoted apoptosis of tumor cells by silencing protein synthesis through posttranscriptional regulation^25^. Collectively, these findings suggest that METTL13 could be a crucial gene of tumorigenesis and development.

In the present study, we examined the expression of METTL13 in paired HNSCC and para-carcinoma samples, and found METTL13 was significantly increased in HNSCC. Further study confirmed it was related to overall survival. And METTL13 knockdown significantly inhibits the proliferation and induced apoptosis in vitro. Conversely, the overexpression of METTL13 can promote malignant phenotypes of tumor. By RNA-seq, we figured out that METTL13 regulated the EMT pathway. In terms of mechanism, we managed to demonstrate that METTL13 could enhance translation efficiency of Snail, the key factor in EMT, hence regulating the progression of EMT. Therefore, our data revealed the function and underlying mechanism of METTL13 in HNSCC, providing both a prognostic marker and drug candidate for this malignancy.

## Results

### METTL13 expression was upregulated in HNSCC and negatively correlated with clinical survival

**Fig. 1:**
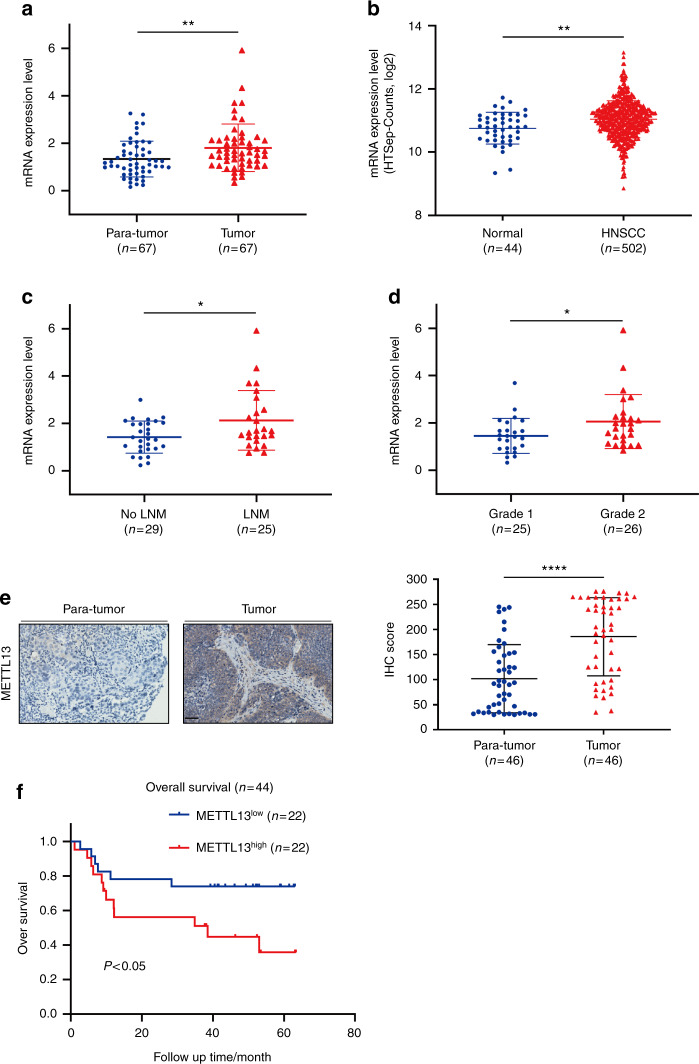
The expression of METTL13 is increased in HNSCC and its high expression is correlated with poor prognosis. **a** Real-time PCR analysis of METTL13 mRNA level in 67 pairs HNSCC tumor tissues and paired adjacent nontumor tissues. The METTL13 mRNA levels are normalized to the β-actin RNA levels. **b** Scatter diagram comparing levels of METTL13 mRNA in normal tissues and HNSCC tissues in published data from TCGA database, **P < 0.01 by *t*-tests. **c** The relationship between METTL13 expression and lymph node metastasis in patients with HNSCC (*n* = 54). **d** The relationship between METTL13 expression and HNSCC tumor grade (*n* = 54). **e** IHC staining of METTL13 in HNSCC and paired adjacent nontumor tissues and the IHC score quantification. Scale bar, 50 μm. **f** Kaplan–Meier analysis of overall survival of patients (*n* = 44). Patients were divided into METTL13 low group and METTL13 high group according to the real-time PCR results

To investigate the expression pattern of METTL13 in HNSCC, we firstly analyzed its mRNA expression level by qPCR in 67 HNSCC and paired para-carcinoma tissues obtained from Hospital of Stomatology of Sun Yat-sen University. Notably, significant upregulation of METTL13 was observed (*P* < 0.001, Fig. 1a). Consistently, TCGA analysis data also showed that METTL13 was upregulated in the HNSCC samples (Fig. 1b). In addition, upregulation of METTL13 in HNSCC patients was significantly associated with the clinical indexes including lymph node metastasis and histopathological grading (Fig. 1c, d). Next, we examined the METTL13 expression at protein level by immunohistochemistry (IHC). Higher expression of METTL13 was observed in human HNSCC samples than paired normal tissues (Fig. 1e). To further explore the potential prognostic value in HNSCC, a total of 44 patients were segmented into METTL13 low and high expression groups based on the qPCR results. The data showed METTL13 significantly negatively correlated with overall survival of HNSCC patients (*P* < 0.05, Fig. 1f). Collectively, we showed that the expression of METTL13 was upregulated in HNSCC, and it might play a key role in HNSCC development and progression.

### Knockdown of METTL13 inhibited HNSCC cell malignant phenotypes in vitro

**Fig. 2:**
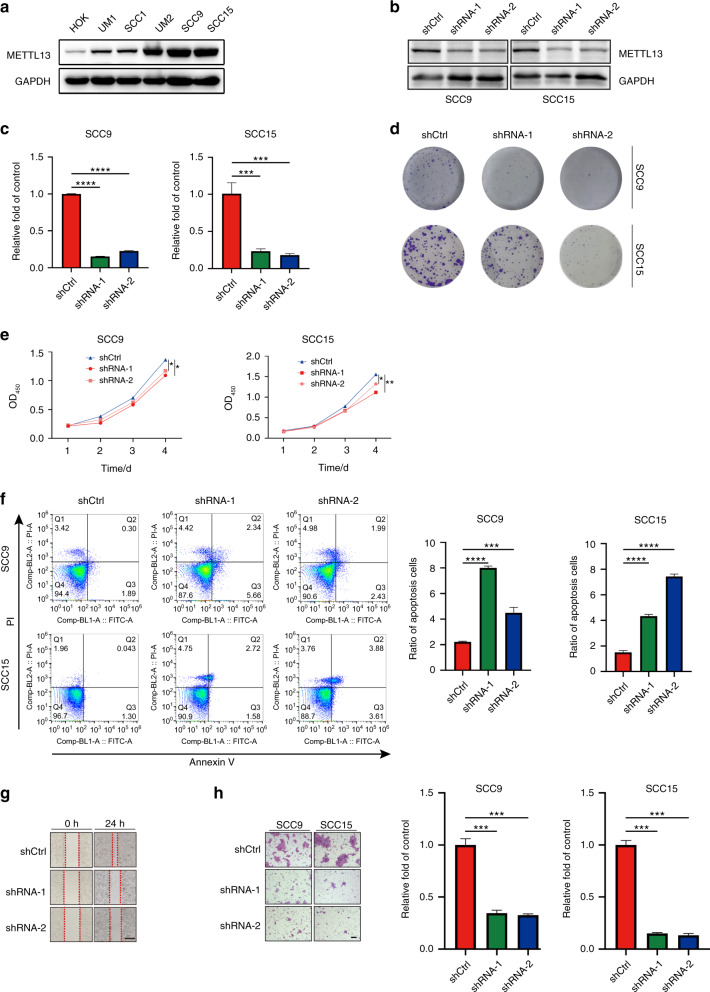
Knockdown of METTL13 attenuates cell proliferation in vitro. **a** Western blot analysis of METTL13 expression in different HNSCC cells. GAPDH was served as internal control. **b**, **c** Knockdown efficiency of METTL13 in SCC9 and SCC15 cells was confirmed by western blot and real-time PCR analysis. **d** The colony formation assay was performed on SCC9 and SCC15 cells transfected with LV-shRNA-1, LV-shRNA-2, and LV-shCtrl, colony numbers were measured after crystal violet staining. **e** The CCK-8 assay examining the effect of METTL13 decrease on the proliferation of SCC9 and SCC15 cells. The experiments were performed in triplicate. **f** Cell apoptosis rate of SCC9 and SCC15 cells stable silenced METTL13 expression was detected by flow cytometry. Ratio of early and late apoptotic cells was collected and presented in the column chart. **g** Knockdown of METTL13 decreased cell migration capacity, as determined by the wound-healing assay. Scale bar, 100 μm. **h** Cell migration abilities of SCC9 and SCC15 cells stably transfected with vector or shRNA were evaluated by transwell migration assay. Scale bar, 100 μm

Aberrant cellular activities often occur in cancer cells, such as continuous proliferative, evading growth inhibition, resisting cell apoptosis, and active invasion and metastasis^26^. To explore the functional role of METTL13 in HNSCC cells, we first selected six HNSCC cell lines and compared their METTL13 expression by the western blotting assay (Fig. 2a). The results revealed higher METTL13 protein level in two HNSCC cancer cell lines (SCC9 and SCC15) and lower METTL13 protein level in SCC1 cell line (Fig. 2a). Hence, we chose SCC9, SCC15, and SCC1 cell lines for further functional study. We then generated stable METTL13-knockdown SCC9 and SCC15 cell lines using shRNA targeting METTL13 and confirmed the successful knockdown of METTL13 gene at protein and mRNA levels (Fig. 2b, c). Next, we performed clone formation assay and CCK-8 assay to explore the effect of METTL13 gene knockdown on the proliferation in HNSCC cell lines. The results showed that the depletion of METTL13 significantly not only inhibited the clone formation ability but also inhibited cell growth rate in these two cell lines (Fig. 2d, e). Next, we utilized a flow cytometer to study the role of METTL13 in cell apoptosis. Our results demonstrated a higher apoptosis rate after ablation of METTL13 in HNSCC cells (Fig. 2f). Subsequently, wound-healing assay results revealed that silencing of METTL13 significantly suppressed cell migration and prolonged the repair time (Fig. 2g). Transwell cell migration assay further proved knockdown of METTL13 attenuated the metastasizing ability of HNSCC cells (Fig. 2h). Taken together, these results demonstrated that METTL13 is critical for proliferation, apoptosis and invasion of HNSCC cell lines.

### METTL13 downregulation negatively correlates with HNSCC CSC-like properties

**Fig. 3:**
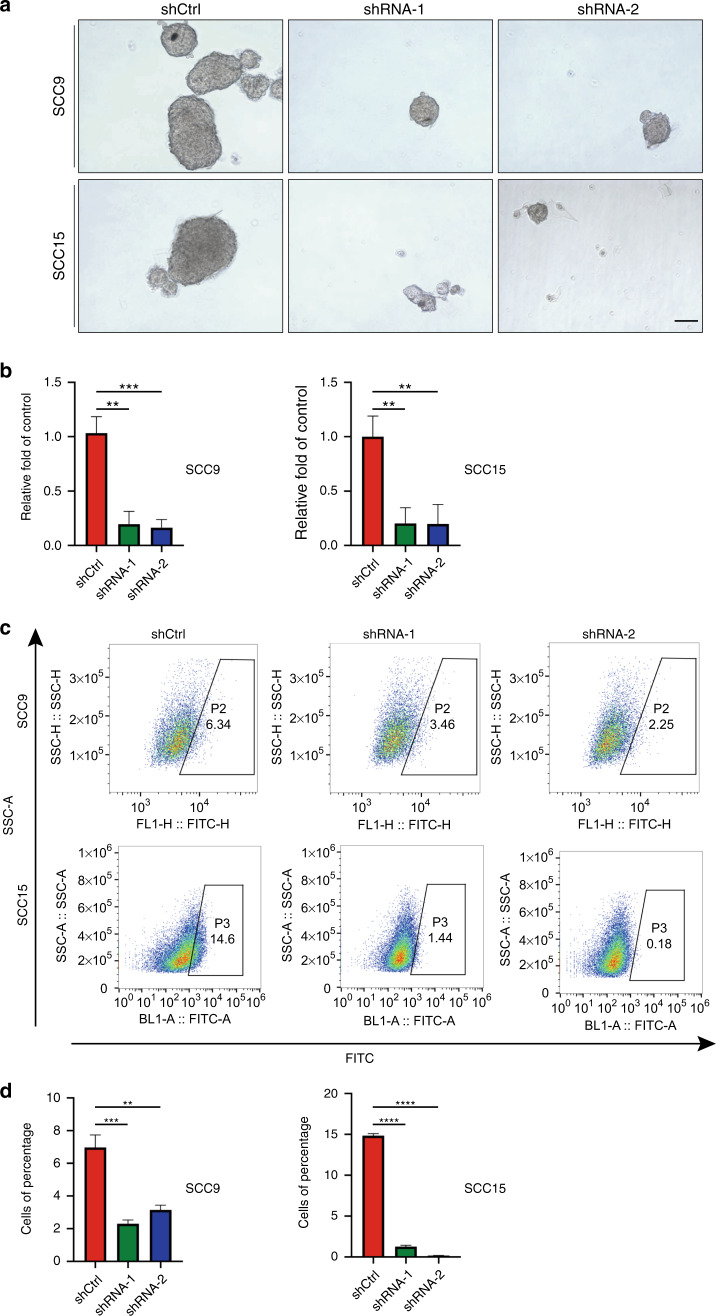
METTL13 was demonstrated to be associated with HNSCC CSC-like properties. **a**, **b** Representative images and quantification results of sphere-forming assay for SCC9 and SCC15 transfected with LV-shRNA-1, LV-shRNA-2, and LV-shCtrl. **c** Flow cytometry analysis of ALDH activity of SCC9 and SCC15 cells. **d** Column chart of cells with ALDH activity

We also investigated whether METTL13 played a role in HNSCC CSCs like properties. First, we determined HNSCC cellular self-renewal capacity by conducting the sphere-forming assay. Downregulation of METTL13 greatly reduced the size and number of tumor spheres when compared with those formed by control cells in both SCC9 and SCC15 cell lines (Fig. 3a, b), indicating that their ability to self-renew was impaired. We then further assessed the CSC-like properties of METTL13 using assay to detect aldehyde dehydrogenase (ALDH) activity, which was regarded as a reliable marker for CSCs^27^. As shown in Fig. 3c, d, ALDH activity was significantly reduced in cells treated with sh-METTL13. Therefore, METTL13 played an essential part in maintaining HNSCC cells self-renewal ability.

### Overexpression of METTL13 could promote HNSCC tumorigenicity

**Fig. 4:**
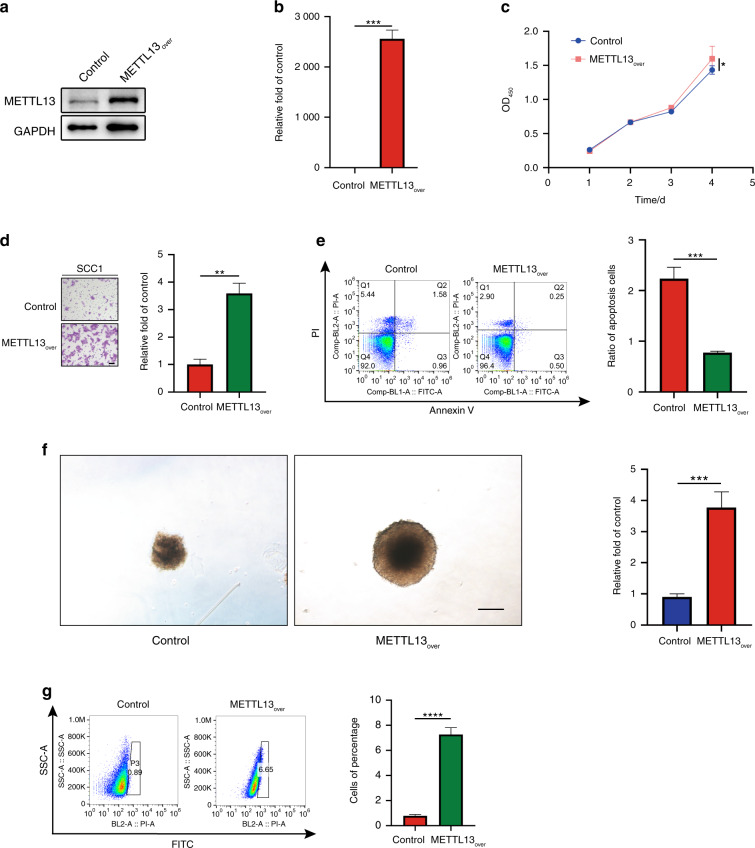
Overexpression of METTL13 promotes HNSCC cell malignant phenotype. **a**, **b** The expression levels of METTL13 in SCC1 METTL13-overexpressing cells or corresponding negative control were confirmed by western blot and real-time PCR analysis. **c** The CCK-8 assay examining the effect of METTL13 overexpression on the growth of SCC1 cells. **d** Cell migration abilities of SCC1 cells with METTL13 overexpression were evaluated by transwell migration assay, left; right, column chart. Scale bar, 100 μm. **e** Cell apoptosis rate was examined by flow cytometry using the annexin V apoptosis detection kit. Ratio of early and late apoptotic cells was collected and presented in the column chart. **f** Representative images and quantification results of sphere-forming assay for SCC1 cell lines. **g** ALDH activity was detected by the flow cytometry to examine the effect of METTL13 overexpression on the cellular stemness

To further explore the function of METTL13 in HNSCC, a stable METTL13 overexpression SCC1 cell line was generated. The upregulation efficiency was verified by western blotting and qPCR assays (Fig. 4a, b). The CCK-8 assay revealed overexpression of METTL13 remarkably increased the capacity of HNSCC cells to proliferate (Fig. 4c). Next, we found that extra acquisition of METTL13 could accelerate cell migration of SCC1 in the transwell assay (Fig. 4d). Consistently, lower rate of apoptotic cells was captured in METTL13_over_ group by the flow cytometry (Fig. 4e). Furthermore, METTL13_over_ group tended to form larger size of tumor spheres after having cultured for 7 days (Fig. 4f). On the other hand, overexpression of METTL13 endowed SCC1 cells with higher ALDH activities (Fig. 4g). To sum up, these data show that METTL13 plays a potential oncogenic role by increasing or inducing the malignant proliferation, migration, and CSC-like properties in HNSCC cells.

### METTL13-regulated EMT signaling pathway by enhancing translation efficiency of Snail to facilitate HNSCC progress

**Fig. 5:**
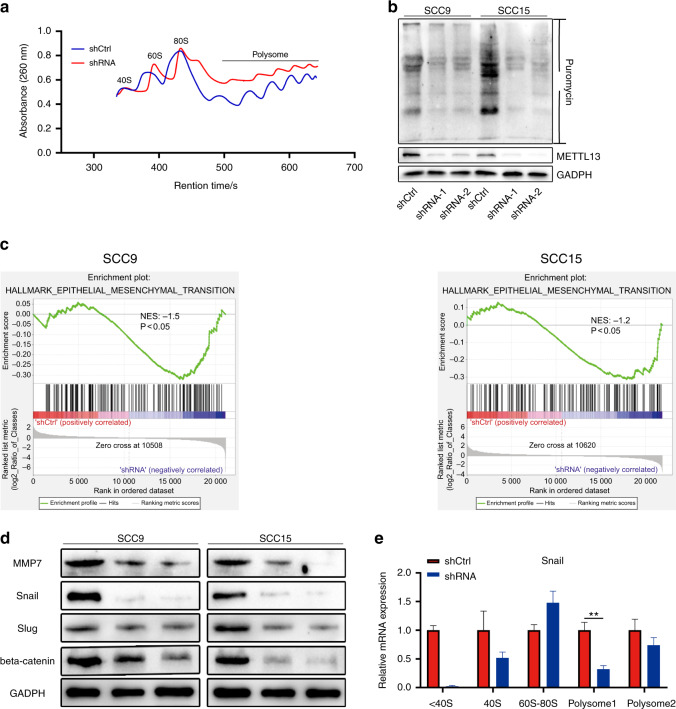
METTL13 promotes translation of Snail to regulate EMT in HNSCC cancer cells. **a** Polysome profiling of wild-type (shCtrl) or Mettl13 knockdown (shRNA) was analyzed. **b** SUnSET assays were used to confirm METTL13 decrease could suppress translation of HNSCC cells. **c** Representative GSEA curves for significant enriched gene sets related to EMT pathways upon METTL13 silencing compared to shCtrl cells. **d** Western blot analysis of EMT relevant molecules in METTL13-knockdown cell lines. GAPDH is used as an internal control. **e** Analysis of Snail mRNA in nontranslation segment (<40S), 40S, 60S, 80S, and polysome for the Mettl13-knockdown cells compared to control cells. β-actin was functioned as internal control

We have demonstrated that METTL13 expressly promoted HNSCC proliferation and metastasis, but the specific mechanism was unclear. Previous studies have reported that METTL13-mediated methylation of eEF1A promotes oncogenesis via increased translation elongation and protein synthesis in cancer cells^19^. And indeed, our polysome profile assay results revealed that decrease of METTL13 suppressed total protein translation in HNSCC cell (Fig. 5a). In addition, the surface sensing of translation (SUnSET) monitoring method confirmed protein synthesis was inhibited after METTL13 depletion (Fig. 5b).

Previous study has indicated that METTL13 is involved in RAS-driven cancer development, we wonder whether depletion of METTL13 in HNSCC is also related to RAS signaling pathway^19^. Therefore, RNA sequencing was performed using the METTL13 control and knockdown groups in both SCC9 and SCC15 cell lines. Interestingly, results from Gene Set Enrichment Analysis (GSEA) indicated that EMT signaling pathway was significantly negatively correlated with the downregulation of METTL13 in HNSCC cell lines (Fig. 5c), suggesting that downregulation of protein synthesis might prone to EMT-related factors. To examine whether this is the case, a series of key proteins associated with EMT pathway were examined by western blotting (Fig. 5d, data not shown). Notably, as a key transcription factor in EMT, Snail was significantly downregulated in METTL13-deficient cells (Fig. 5d). Therefore, we hypothesized that METTL13 could regulate EMT signaling pathway by regulating the translation activity of Snail mRNA. To test that, we performed polysome profile assay using sucrose gradient centrifugation to separate the ribosome and polysome segments. Based on these results, we used qPCR to detect the distribution of METTL13 affected mRNAs in the polysome segment. And the results showed Snail mRNA was significantly downregulated in the METTL13-deficient groups in the polysome levels (Fig. 5e), indicating that the METTL13 is required for translation of Snail, which consistent with our previous conjecture. Altogether our results support that Snail is involved in METTL13-regulated EMT in HNSCC, and METTL13-regulated EMT signaling pathway by enhancing translation efficiency of Snail.

### Decreased METTL13 expression weakened malignant phenotypes in vivo

**Fig. 6:**
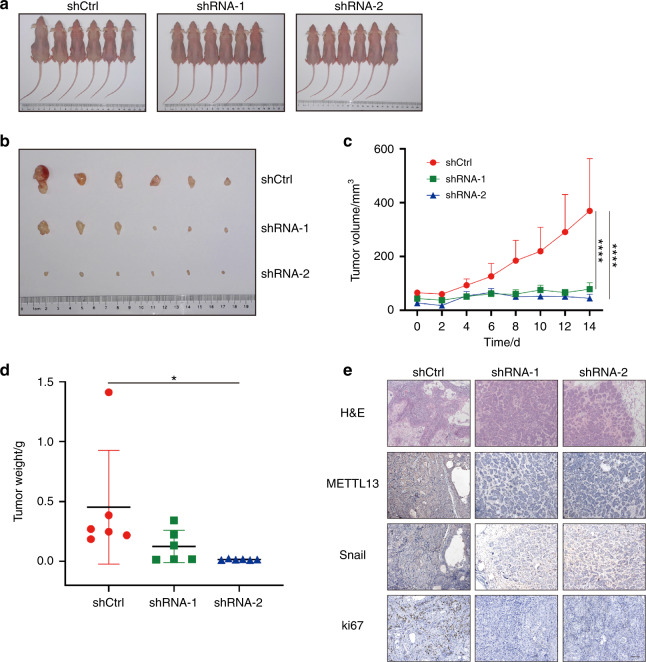
METTL13 regulates HNSCC cell proliferation in vivo. **a** BALB/C nude mice (*n* = 6) were subcutaneously transplanted with SCC15 cells transfected with LV-shRNA-1, LV-shRNA-2, and LV-shCtrl. **b** Tumors were collected after 4 weeks. **c** Tumor volume was calculated every 2 days and determined by length × width^2^/2. **d** The tumor weigh was measured after sacrificing mice. **e** Representative images of H&E and immunohistochemistry (IHC) detection of METTL13, Snail, and Ki67 in xenograft studies. Scale bar, 200 μm

Xenograft studies were then conducted to investigate whether METTL13 could recapitulate the in vitro malignant phenotypes in vivo. Control and two METTL13 shRNA SCC15 cell lines were subcutaneously injected into nude mice. Mice were sacrificed after 4 weeks. In accordance with our in vitro results, deficiency of METTL13 in SCC15 cell dramatically suppressed tumor growth when compared with the control in vivo (Fig. 6a). And the tumor volume and tumor weight results further confirmed this effect (Fig. 6b, c). In order to get a more profound understanding of the function of METTL13 in vivo, we obtain the tumor sections. Next, hematoxylin and eosin (H&E) staining and METTL13 IHC results verified that METTL13 expression was decreased in sh-METTL13 tumor cells compared to the control (Fig. 6d, e). The staining of Ki67, a proliferation marker, showed alleviated cell growth after METTL13 gene knockdown, which was consistent with in vitro results (Fig. 6e). Consistently, IHC staining of nude mice showed the expression of Snail protein was reduced by the silencing of METTL13 (Fig. 6e). Collectively, these data demonstrated that METTL13 is required for tumorigenic ability of HNSCC cells in vivo.

### METTL13 promotes the progression of HNSCC by targeting Snail

**Fig. 7:**
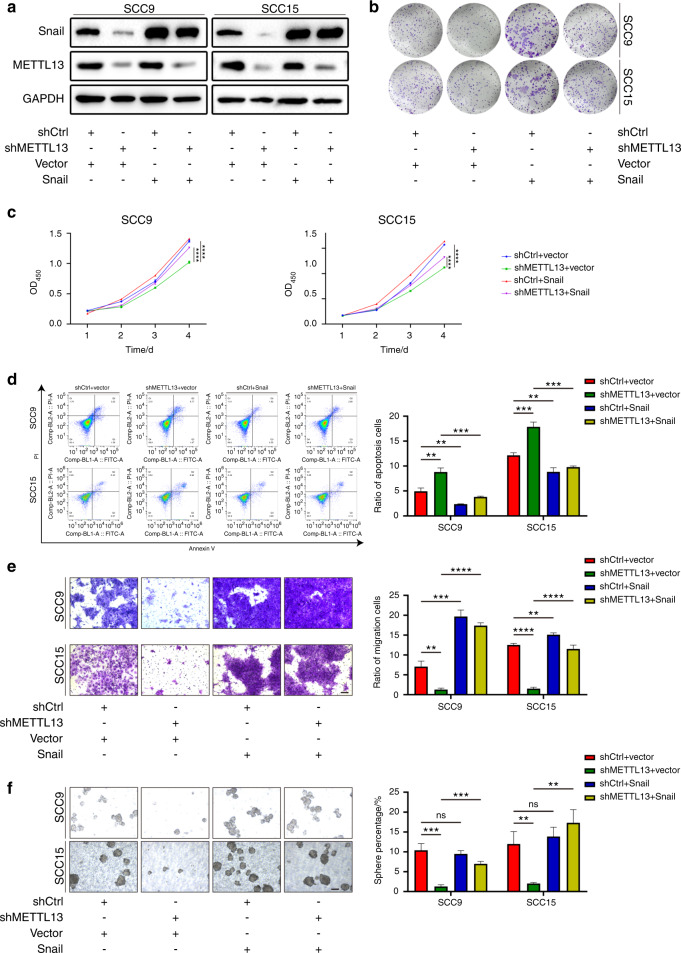
METTL13 promotes the progression of HNSCC by targeting Snail. **a** Snail was overexpressed in METTL13-depleted SCC9 and SCC15 cells, Snail, METTL13, and GAPDH level is examined by western blot. **b** The growth ability is examined by the clone formation assay. **c** Snail is upregulated in METTL13-knockdown cells, CCK-8 assay reveals that the suppressive effect of METTL13 on HNSCC cell growth is rescued. **d** Apoptotic cell ratio is tested by annexin V-FITC/PI kit. The stained cells are analyzed by flow cytometry. **e** Snail was overexpressed in METTL13-depleted SCC9 and SCC15 cells, transwell experiments overexpression of Snail can restore cellular migration ability. Scale bar, 100 μm. **f** Sphere formation assay revealed that the suppressive effect of METTL13 on sphere-forming ability was restored. Scale bar, 100 μm

We have found that METTL13 promoted the proliferation, invasion, and metastasis of HNSCC cells in vitro and in vivo via regulating the EMT pathway and Snail was the downstream candidate. To further investigate whether Snail was the main downstream mediator of METTL13, we overexpressed Snail in both control and METTL13-knockdown cells (Fig. 7a). Clone formation assay and CCK-8 assay showed that overexpression of Snail could significantly rescue the clone formation ability and cell proliferation inhibited by METTL13 knockdown (Fig. 7b, c). Consistently, the higher apoptosis rate of HNSCC cells caused by inhibiting METTL13 was rescued by Snail overexpression (Fig. 7d). Meanwhile, the transwell assay demonstrated inhibition on HNSCC cells migration by ablation of METTL13 was weakened in Snail overexpressing cells (Fig. 7e). In addition, Snail also significantly rescued the sphere formation ability in SCC9 and SCC15 cell lines after METTL13 was downregulated (Fig. 7f). In summary, these results indicated that Snail functions as an effector in METTL13 induced promotion of progression in HNSCC.

## Discussion

Translation is one of the most energy consumption processes inside the cell and dysregulation of translation mechanisms is a common cause of neoplastic diseases^28,29^. Because in cancer cells where mRNA translation is usually highly active, it is necessary to increase the rate of protein synthesis^14^. And increasing evidences revealed translation elongation factor modification could cause promotion or inhibition of protein synthesis^30–33^. In our study, we demonstrated that METTL13, a regulator of translation elongation, showed higher expression levels in HNSCC tumors than adjacent normal tissues and might serve as a prognostic marker for overall survival in HNSCC patients.

METTL13 has been reported to be involved in cell apoptosis, stem cell biology, and various cell signal and metabolic pathways^34^. However, whether it participates in tumorigenesis activity in HNSCC remains largely elusive. Here, we documented a detailed oncogenic role of METTL13 in HNSCC cell lines. We found that METTL13 is necessary and required for proliferation and EMT of HNSCC cancer cells both in vitro and in vivo. The translation level of METTL13-depleted HNSCC cells underwent significantly decrease compared with control cells. Intriguingly, GESA analysis of the transcriptome showed that genes downregulated in the METTL13-depleted HNSCC are related to EMT process. Previously, METTL13 has been shown to promote cell proliferation and metastasis by elevating the levels of TCF3 and ZEB in hepatocellular carcinoma^35^. In addition, METTL13 can participate in development of Ras-dependent epithelial tumors of the pancreas and lung^19^. In HNSCC, we found depletion of METTL13 profoundly reduced the protein level of Snail, one of the key transcription factors of EMT. More specifically, we demonstrated that METTL13 could enhance the translation efficiency of Snail mRNA to promote EMT and HNSCC progression. It is highly possible that the inhibition of the metastatic ability of cancer cells after deletion of METTL3 may be mitigated by Snail’s overexpression. Moreover, the present study found that the Snail served as a necessary effector of oncogene function of METTL13 in HNSCC. Previous studies revealed that EMT could be utilized by the tumor cells to perform metastatic cascades^7,36^, and scientific groups began to work on the its underlying mechanisms in tumor progression. In one study, EMT signaling pathway was blocked through modulation of the key protein translation in breast cancer^37^. Thereby, our study provided a more profound understanding of EMT in HNSCC.

In conclusion, our data revealed the tumor promoter function of METTL13 in HNSCC. METTL13 could regulate the progression of EMT by promoting Snail mRNA translation. These results suggested that METTL13 might function as a promising drug target. METTL13 could regulate the translation of downstream proteins by affecting the stability of 80S ribosome structure. Although deeper researches are needed to uncover the mechanism of METTL13 carcinogenesis and as a drug target for HNSCC in the future, our present research has built the foundation for better therapeutic strategies.

## Materials and methods

### Cell culture and patient samples

SCC9, SCC15, and SCC1 cell lines were obtained from Type Culture Collection of the Chinese Academy of Sciences in Shanghai, China. And all the cells were cultured in Dulbecco’s modified Eagle’s medium (DMEM, Gibco, 11885-076) and supplemented with 10% fetal bovine serum (FBS, Gibco, 10270-106) and 1% penicillin–streptomycin (Gibco, 15140122) at a 37 °C incubator containing 5% CO_2_.

Both HNSCC tissues and adjacent tissues were collected from HNSCC patients who underwent surgery at the Hospital of Stomatology, Sun Yat-sen University. This study received the informed written consent from all patients before the experiment.

### Cell transfection

METTL13 gene expression was inhibited by two sequences of shRNAs, shRNA-1: AATGTGGACCTTCATTCGGTC and shRNA-2: TTGTGAGACACATCCTTCAGC. The synthesized and purified METTL13 gene and control fragment were imported into a lentivirus vector. And recombinant lentivirus was produced from 293T cells by Lipofectamine^TM^ 2000 transfection reagent (Thermo Fisher Scientific, 11668019), and transfected into HNSCC cell lines by polybrene (8 μgmL^−^^1^). And pFLAG-CMV2-METTL13 plasmid and puro-Snail plasmid were used for overexpression of METTL13 and Snail.

### Total RNA extraction and real-time fluorescent quantitative PCR

The total RNA of cells was obtained according to the instructions provided by the TRIzol reagent (Invitrogen, 15596026). Next, TB Green™ Premix Ex Taq II kit (Takara, RR820B) was used for real-time PCR according to the instructions and operated under the Bio-Rad CXF96 real-time system (Bio-Rad, USA). β-actin functioned as an internal control to calculate the relative quantity. The primers pair used for real-time PCR was as follows: METTL13 (forward): 5′-CAGGAGGTTGATTACAGTGG-3′; METTL13 (reverse): 5′-CTCCATGACTCTAGCCGACA-3′. β-actin (forward): 5′-GATCATTGCTCCTCCTGAGC-3′; β-actin (reverse): 5′-ACTCCTGCTTGCTGATCCAC-3′. Snail1 (forward): 5′-TGCCCTCAAGATGCACATCCGA-3′; Snail1 (reverse): 5′-GGGACAGGAGAAGGGCTTCTC-3′.

### Total protein extraction and western blotting analysis

The total protein of HNSCC cell lines was harvested with ice-cold radioimmunoprecipitation assay (RIPA) lysis solution. And the cell protein component was isolated by 12% SDS polyacrylamide gel electrophoresis and transferred to the polyvinylidene fluoride membrane (Millipore, Germany). Enhanced chemiluminescence reagents were used to detect the protein antibody complexes. And the following primary antibodies were used: Snail (Cell Signaling Technology, 3879T), Twist (Cell Signaling Technology, 46702S), Slug (Cell Signaling Technology, 9585T), MMP7 (Abcam, ab216631-100 µL), MMP9 (Abcam, ab74277-100 µL), E-cadherin (Affinity, AF0131-100), N-cadherin (Affinity, AF4039-50 µL), vimentin (Abcam, ab24525-50 µL), beta-catenin (Affinity, AF6679-100), and METTL13 (GeneTex, GTX120626-S). GADPH (Proteintech, 10494-1-AP) was served as internal control.

### Colony formation assay

Two-hundred cells were added into six-well plates and were cultivated for about 2 weeks. Cells were fixed for 30 min after 14 days with 4% paraformaldehyde and stained with 0.1% violet crystal. Each group had been repeated in triplicate.

### Cell proliferation assay

Cell proliferation ability was assessed via CCK-8 assay (Dojindo, CCK8-500). According to the standard procedure, 100 μL cell suspension containing 2 × 10^3^ cells was seeded into each well of the 96-well plate and cultured in a cell incubator. At equal time intervals (four times every 24 h), 10 μL reaction agent was added to each well and cultured for 1 h at 37 °C, and then taken out for absorbance value detection at 450 nm (Infinite 200 PRO).

### Wound-healing and cell migration assay

For the wound-healing experiment, cells were transplanted and grown until they developed a monolayer of 90% fusion. Cells were then removed with the tip of an aseptic straw and treated in FBS-free medium according to the instructions. The scratched visual field was randomly selected under the microscope, and the migration distance of the cells to the scratched area was measured.

Transwell system was utilized for migration analysis. And DMEM containing 10% FBS was served as a chemical inducer. Cells were fixed with 4% paraformaldehyde at 4 °C for 30 min after 18 h of incubation at 37 °C and 5% CO_2_. Cells were stained with crystal violet after rinsing with PBS and incubated for 45 min at room temperature. The cells on the cell culture insert’s upper side were erased with a cotton swab, and washed thoroughly with PBS. The average number of cells was calculated according to the random visual field. Each sample was counted for three chambers.

### Flow cytometric analysis of annexin V apoptosis

Cell apoptosis rate was detected using flow cytometry as per the annexin V apoptosis detection kit (Dojindo, AD10) instructions. HNSCC cell lines were transplanted into six-well plates (1 × 10^5^ mL^−1^ per well) for apoptosis analysis and cultivated at 37 °C for 48 h in complete medium. Cells were then harvested and stained with the kit after the treatment with non-EDTA trypsin. Cell apoptosis assay was performed on a flow cytometer with BD FACSDiva software 6.0 (BD Biosciences, New York, NY, USA). All the experiments were repeated at least three times.

### Flow cytometric analysis of ALDH activity assay

ALDH activity assay was conducted by the ALDEFLUOR fluorescence reagent system (Stem Cell Technology, 01700). In short, we labeled each sample with a test tube and a control tube. The cells were lysed and collected into the test tube and activated ALDEFLUOR reagent was added to each cell suspension. Half of the cell fluid in the test tube was added to the control tube, 5 μL diethylaminobenzaldehyde was immediately added to the control tube, and then the fluid was incubated at 37 °C for 45 min and centrifuged. Then, the supernatant was removed and resuspended in the determination buffer. Finally, the analysis was performed on the flow cytometry.

### IHC staining and H&E staining

IHC and H&E staining procedure had been described previously^38^. All the primary antibodies including METTL13 (GeneTex, GTX120626-S), Snail (Cell Signaling Technology, 3879T), and Ki67 (Affinity, AF0198) were used at dilution in 1:100. IHC staining intensity was scored as 0 (negative), 1 (weak), 2 (medium), or 3 (strong). And IHC score was defined by multiplying the percentage of positive cells (P) by the intensity (*I*), formula: Q = P × *I*; maximum = 300.

### RNA sequencing

SCC9 and SCC15 cells transfected with shCtrl, shRNA-1, and shRNA-2 were sent for RNA sequencing. Briefly, the total RNA was extracted using TRIzol as mentioned previously. RNA quality and purity were checked by Nano 300.

### SUnSET assay

As for translation analysis, the cells were incubated with puromycin (Beyotime, ST551-50 mg) of 1 µmol · L^−1^ final concentration at 37 °C for 30 min. After that the cells were collected and lysed in RIPA buffer containing with 1 mmol · L^−^^1^ PMSF and protease inhibitor. The whole cell lysate of 5–10 μg was detected by western blotting with anti-puromycin primary antibody (Sigma-Aldrich, MABE343).

### Polysome profiling and fractionation

Polysome profiling assay was conducted as described previously^39^. In short, cells with control or METTL13 decreased were cultured in 15 cm dishes (about 1 × 10^7^ cells in each dish). Then, cells were loaded with actinomycin at 100 μg · mL^−1^, then incubated for 2 min at 37 °C. Then, cells were collected and cell extract was loaded on a sucrose gradient of 10–50% and prepared with a BioComp gradient station. The gradient was rotated at 36 000 r · min^−1^ and 4 °C for 2 h in the TH-641 rotor (Sorvall). The gradient (260 nm) was analyzed and the components were collected by the BioComp gradient station.

### Sphere formation assays

According to the method we used before^40^, the stem cell culture medium containing 3 × 10^3^ cells per mL will be put into the six-well ultralow attachment plates, and will be incubated for 7–10 days in a 37 °C incubator containing 5% CO_2_. The stem cell culture medium was replaced every 2 days. After spheres were formed, they were digested into single sphere by 0.25% trypsin-EDTA. Photographs and counts were taken under a microscope.

### Animal studies

For the in vivo subcutaneous transplanted model studies, 4-week-old BALB/C female nude mice obtained from Center of Experimental Animal of Sun Yat-sen University were divided into three groups (*n* = 6 per group) randomly. HNSCC cell lines transfected with METTL13-deficient or control plasmid were subcutaneously injected into mice to explore the effect in vivo. After 4 weeks, all the mice were sacrificed and we resected xenograft tumors intactly. The volume of transplanted tumor volume was determined as follows: length × width^2^/2.

### Statistical analysis

The experiments had been replicated at least three times. The sample size necessary for vivo studies to discern difference in statistical significance is at least six animals in each group.

Data were shown as mean ± SD. All the mathematical analysis of the data was done by GraphPad Prism 8.0. Significance levels are: ns denotes not significant, *P < 0.05, **P < 0.01, ***P < 0.001, and ****P < 0.000 1.
